# The Spatial Selective Auditory Attention of Cochlear Implant Users in Different Conversational Sound Levels

**DOI:** 10.3390/jcm10143078

**Published:** 2021-07-12

**Authors:** Sara Akbarzadeh, Sungmin Lee, Chin-Tuan Tan

**Affiliations:** 1 Department of Electrical and Computer Engineering, University of Texas at Dallas, Richardson, TX 75080, USA; Chin-Tuan.Tan@utdallas.edu; 2 Department of Speech-Language Pathology and Audiology, Tongmyong University, Busan 48520, Korea; Slee18@tu.ac.kr

**Keywords:** cochlear implant, cocktail party scenario, selective auditory attention, speech recognition

## Abstract

In multi-speaker environments, cochlear implant (CI) users may attend to a target sound source in a different manner from normal hearing (NH) individuals during a conversation. This study attempted to investigate the effect of conversational sound levels on the mechanisms adopted by CI and NH listeners in selective auditory attention and how it affects their daily conversation. Nine CI users (five bilateral, three unilateral, and one bimodal) and eight NH listeners participated in this study. The behavioral speech recognition scores were collected using a matrix sentences test, and neural tracking to speech envelope was recorded using electroencephalography (EEG). Speech stimuli were presented at three different levels (75, 65, and 55 dB SPL) in the presence of two maskers from three spatially separated speakers. Different combinations of assisted/impaired hearing modes were evaluated for CI users, and the outcomes were analyzed in three categories: electric hearing only, acoustic hearing only, and electric + acoustic hearing. Our results showed that increasing the conversational sound level degraded the selective auditory attention in electrical hearing. On the other hand, increasing the sound level improved the selective auditory attention for the acoustic hearing group. In the NH listeners, however, increasing the sound level did not cause a significant change in the auditory attention. Our result implies that the effect of the sound level on selective auditory attention varies depending on the hearing modes, and the loudness control is necessary for the ease of attending to the conversation by CI users.

## 1. Introduction

Selective auditory attention is the ability of the auditory system to attend to a target sound source and ignore the competing sounds in multi-speaker environments, known as the cocktail party scenario. Spatial, temporal, and frequency cues help to identify and separate the speech streams. If these cues are not accessible in the auditory pathway, this may result in decreased spatial selective auditory attention and speech intelligibility [1,2].

Previous studies have shown that spatial selective auditory attention is degraded by hearing loss [1,2,3,4,5,6]. Most hearing-impaired (HI) listeners suffer from poor spatial hearing, especially when they encounter social settings where multiple people talk in a group. This is particularly the case for cochlear implant (CI) listeners who have severe hearing loss, and their performance falls much below that of the normal hearing (NH) listeners in spatial hearing tasks [7,8,9,10]. Besides the intelligibility of the target stimulus, which is commonly used to explain the degraded auditory selective attention, localizing the target speech source among distractors can also concurrently contribute to the difficulty faced by CI users in spatial hearing. Poor temporal resolution typically experienced by CI users [11] prevents them from taking advantage of the interaural time difference (ITD) cues [12]. In addition, CI users are also less sensitive to changes in levels [13], implying that they could not benefit as much as NH listeners from the interaural level difference (ILD) cues for sound localization.

In the last few years, some studies [2,5,6,14,15,16] have examined the different factors that affect the spatial selective auditory attention with hearing-impaired listeners. One way of examining spatial hearing with headphones is to determine where a sound is coming from, which is known as lateralization. Strelcyk and Dau [17] investigated the effect of stimuli level on the sound lateralization for NH and HI listeners. They measured the lateralization threshold for 750 Hz tones, fixed at 70 and 35 dB SPL. The results showed that generally, the lateralization threshold improved at the higher stimulus level comparing to the lower stimulus level. The difference between NH and HI listeners’ performance at the lower tone level was smaller than that at the higher tone level. Consistent with this result, Smoski [18] observed a smaller deficit in HI listeners’ lateralization (relative to NH) at a lower tone level. However, HI listeners in the study by Hawkins and Wightman [19] showed a smaller lateralization deficit at a higher stimulus level than that at a lower stimulus level, when the narrow-band noise was used as stimulus in a quiet condition. The different outcomes of these studies [18,19] may be attributed to the type of stimuli (tone/noise) used in these studies. Strelcyk and Dau explained that at higher stimulus levels, lateralization judgments could arise from the excitation of a larger area of the basilar membrane instead of the local excitation area [17]. NH listeners may take advantage of the excitation spread, particularly towards places corresponding to high frequencies, and integrate the additional information placed in high-frequency areas. However, HI listeners with high frequency sensory neural hearing loss may not benefit from this additional information, because it falls in the sloping region of their hearing loss. Nevertheless, the excitation model may not be directly applicable to the cochlear implant participants’ performance.

The spatial separation between the target speech and competing sounds also plays an important role in spatial selective auditory attention. Spatial separation helps listeners segregate sound sources and consequently improves speech intelligibility. The difference between the speech reception threshold (SRT) in co-located and spatially separated sound sources, which is known as spatial release from masking (SRM) [20], is commonly used to measure the benefits of the spatial separation. Gallun et al. [21] compared the SRTs of HI listeners when target sentence levels were presented at 19.5 dB SL and 39.5 dB SL in quiet. In an adaptive approach, the levels of the maskers were adjusted relative to the level of the target sentences to estimate the masked threshold (target-to-masker ratio (TMR) giving 50% correct). The results showed that the masked threshold and SRM improved with an increase in SL, indicating that the listener’s spatial auditory selective attention covaries with the audibility.

The studies mentioned above collectively show evidence that the level of sound does affect the spatial selective auditory attention in both normal-hearing and hearing-impaired listeners. To the best of our knowledge, there is little existing work, but a growing effort in examining the effect of sound level on spatial selective auditory attention in CI users.

Our team [22] has previously shown the effect of speech level on the speech quality as perceived by normal-hearing listeners and CI users. The study showed that even at the same signal-to-noise ratio (SNR), but at different conversational speech levels, different patterns of perceived quality judgment were observed in NH listeners and CI users. While NH listeners preferred higher speech levels, the CI listeners preferred lower speech levels, suggesting that CI listeners choose lower noise levels at the expense of poorer speech audibility. Such evidence of perceptual differences between the two groups inspired us to examine the effect of speech level on the spatial selective auditory attention with NH and CI listeners.

Spatial hearing is commonly assessed from behavioral responses, but it can also be observed in the electrophysiological response. Some studies have also shown that spectral and temporal features of attended speech can be extracted from the cortical response [23,24]. In [25], the electroencephalography (EEG) signal was recorded on the scalps of NH listeners, while they engaged in a two speakers cocktail party scenario. The listeners were instructed to attend to one of the speakers and ignore the other one. A linear decoder was trained to reconstruct the speech envelope using the EEG signal. Listeners’ locus of attention was determined reliably based on the speech envelope reconstructed from the EEG signal. It was shown in [26] that the Spearman correlation between the original speech envelope and the speech envelope reconstructed from the EEG signal increased by increasing the SNR. They demonstrated that the behaviorally measured speech intelligibility was highly correlated with the congruence between the original and reconstructed envelopes. Such an electrophysiological approach was thought to be another applicable measure that drives us to a better understanding of the spatial hearing for NH and CI listeners in this study.

In the current study, we investigated the spatial selective auditory attention in the speech-on-speech masking for NH and CI listeners at different stimulus levels and target azimuths. In order to examine the effect of the speech level independently of the effect of the TMR, target and masker stimuli were presented at different conversational levels while keeping TMR the same. In addition to behavioral experiments, the accuracy of the attended speech envelope reconstruction from the EEG signals was computed as a metric to indirectly measure the selective auditory attention from cortical response in a three speaker cocktail party problem. This study is a preliminary study examining the effect of speech levels on spatial hearing ability, for NH and CI listeners, in both behavioral and neural outcomes. CI listeners with different modes of hearing (bilateral, unilateral, and bimodal) participated in this study. The results were categorized based on the patients’ hearing modes. Due to the limited number of patients in each group and to avoid the biased results due to the unbalanced sample size, the case-by-case results are reported as well.

## 2. Method

### 2.1. Participants

Eight NH listeners (four male; mean age: 24, range: 21–30, SD: 3.5 years) and nine CI users (five male; mean age: 58, range: 24–77, SD: 22 years) participated in this study. Recruitment was made by advertising at local CI patients’ events. CI patients with different modes of impaired/assisted hearing volunteered to participate in the study. All participants self-reported no history of cognitive deficits prior to participation. All NH subjects were verified with 20 dB HL (Hearing Loss) or lower across all octave frequencies between 250 to 8000 Hz in their pure-tone audiograms. Unaided hearing thresholds were identified for the CI group over the same octave frequency range. The demographic information for CI users is shown in Table 1. All procedures were approved by the institutional review board of University of Texas at Dallas. All subjects signed an informed consent form before the experiments, and they were compensated for their participation in the experiments.

### 2.2. Behavioral Experiment

#### 2.2.1. Stimuli

We adopted a matrix sentence test [27] for the behavioral spatial hearing test. On each trial, three spatially separated sentences (one target and two maskers) were presented to the subjects via different loudspeakers. The sentences were formed by concatenating the words, with one word from each of the five categories of the words (name, verb, number, adjective, and noun). Each category consisted of 8 words that were repeatedly spoken by 18 female and 18 male talkers. Table 2 shows the eight words in each of the five categories. The sentences are grammatically correct and sound natural, but conceptually unpredictable to minimize the effect of higher-order language processing.

#### 2.2.2. Procedure

The experiments were conducted in a double-wall soundproof booth. Five speakers placed at −90°, −45°, 0°, 45°, and 90° azimuth were used to present the stimuli. The speakers were located at a radius of 1 m from subjects at the height of the subjects’ heads. A touchscreen monitor was located in front of the subjects.

**Figure 1:**
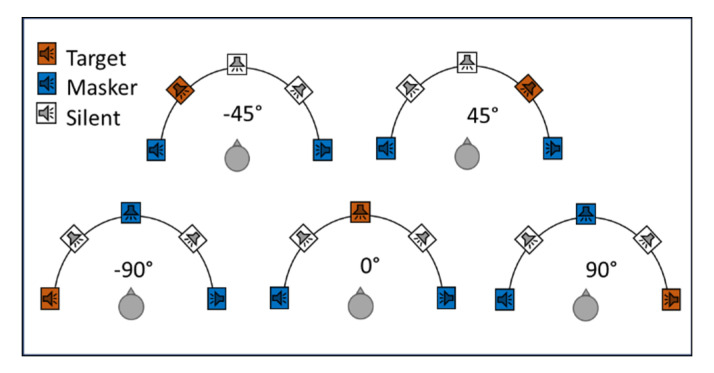
Speakers’ configuration.

In each trial, three different talkers were randomly selected to present the target and masker sentences. Three different sentences were selected from the words listed in Table 2, one as a target and two as maskers. Speech recognition performance for five speaker configurations was examined (Figure 1). To explore the effect of the speech level on spatial selective auditory attention, three different levels at fixed TMR were examined as follows. For CI listeners, the target stimuli were presented at 75, 65, and 55 dB SPL and two maskers each at 65, 55, and 45 dB SPL, respectively. For NH listeners, to avoid ceiling effect, each masker was presented at the same dB SPL level as the target, which means presenting the target at 75, 65, and 55 dB SPL and each of the two maskers at 75, 65, and 55 dB SPL, respectively.

At the beginning of each trial, an arrow appeared on the monitor indicating the location of the target speaker that the subject should attend to. After the subject pressed a start button, stimuli were presented, and the application waited until the subject completed the answer. Once the speech stimuli were presented, the subject had to select the words uttered by the target speaker from a table similar to Table 2 that appeared on the monitor. Subjects were instructed to guess a word if they were not able to identify the word.

The experiment was conducted in three sessions associated with three different stimuli levels, which stretches over a one day visit. At each session, 50 trials (five target speaker configurations × 10 repetitions) were randomly presented to the subject. As mentioned earlier, sentences, words, and talkers were selected randomly for each trial. Before the main experiments, each subject participated in one training session to become familiar with the procedure. At the training session, target stimuli were presented at 65 dB SPL. The maskers were presented at 55 dB SPL for CI listeners and 65 dB SPL for NH listeners.

For bilateral CI users, the experiment was repeated three times; one with both CIs turned on, one with only the left CI turned on, and one with only the right CI turned on. For unilateral CI users who had residual hearing in the contralateral ear, the same experiment was repeated twice; one with CI turned on, and one with CI turned off. For the bimodal CI listener (with hearing aid (HA) in the contralateral ear), the experiment was again repeated three times; one with both CI and HA turned on, one with only CI turned on and HA turned off, and one with CI turned off and only HA turned on.

### 2.3. Electrophysiological Experiment

#### 2.3.1. EEG Recording Setup

The BrainVision system (actiCHamp amplifier) was used to obtain EEG signals via a 64-channel actiCAP Electrode Cap. Scalp electrode placement was set in accordance with the international 10–20 system. The ground electrode was placed at FPz, and the reference electrode was placed at FCz of subject’s head. Horizontal and vertical ocular artifacts were recorded using additional electrodes connected to two bipolar adaptors (BIP2AUX). All electrodes were kept at an impedance lower than 10 kΩ. The EEG signal was digitally recorded using a sampling rate of 1 kHz. The data were stored for offline analysis. Participants were seated in the middle of the sound booth wearing a scalp electrode cap on their heads. They were asked to keep calm, look at a fixed point on the monitor, and minimize eye blinking and muscle movement while the sound was presented.

This study attempted to extract the cortical activities that are entrained to the speech envelope using a decoder. Our EEG experiment consisted of two recording sessions. One session was for collecting data on the speech in quiet to train the decoder, which extracts the speech envelope from the EEG signal. The other session was for measuring the EEG associated with different spatial configuration and sound level conditions in a three-speaker cocktail party scenario. To avoid human fatigue for each participant, the EEG data collection was limited to about 3–4 h over the experiment day.

#### 2.3.2. Recording the Data for Training the Decoder

First, the EEG was recorded with a continuous speech passage and used as the data to train the decoder. The subjects listened to two short story narrations. The first story was “lady or tiger”, narrated by a female speaker (duration = 632.734 s), and the second story was “ambitious guest”, narrated by a male speaker (duration 615.285 s). Both speakers were native American English speakers. Silent gaps (more than 300 ms in duration) were removed from the recordings. The stories were presented at 65 dB SPL from the front speaker without any noise. No question was asked before or after presenting these stimuli. The EEG data were recorded while the subject was listening to the stimuli.

#### 2.3.3. Recording the Data for Testing in the Three-Speaker Cocktail Party Scenario

We presented one target and two maskers to emulate a cocktail party scenario. The stimuli were short passages with a duration of 38 to 45 s. Target and masker passages were selected randomly from the Connected Speech Test (CST) [28] and Speech Intelligibility Rating (SIR) [29] dataset. To remove the effect of the speaker gender, half of the trials had a female target speaker and the other half had a male target speaker. For the trials with a female target, maskers were male, and for the trials with a male target, maskers were female. Three speaker configurations were examined for different permutations of targets and masker locations using the speakers at −90, 0, and 90 azimuths. The stimuli were presented at three different levels with fixed TMR. Targets were presented at 75, 65, and 55 dB SPL and maskers at 65, 55, and 45 dB SPL, respectively. A total of 18 combinations (3 speaker configurations × 2 target genders × 3 speech levels) were randomly presented to each subject as separate trials. At the beginning of each trial, an arrow appeared on the monitor showing the location of the target speech that the subject should attend to. After a one-second pause, the stimuli were presented. After finishing the presentation of each trial, subjects had to respond to two questions about the target passage. The aim of these questions was to keep the subject’s attention to the target speech.

#### 2.3.4. EEG Based Speech Detection Accuracy

**Figure 2:**
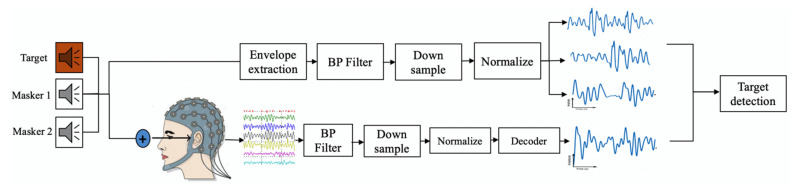
Procedure of the attended speech detection from EEG signal in three-speaker cocktail party test.

The details of the procedure for decoding the attended speech from the EEG signal are presented in Appendix A. With the individualized linear decoder designed for each subject, the speech envelope of the attended speech in the three speaker cocktail party test was reconstructed from the EEG signal. Pearson correlation between reconstructed speech envelope $\hat{s}\left(t\right)$ and each stimulus envelope, target $s_{T}\left(t\right)$, two maskers $s_{M1}\left(t\right)$ and $s_{M2}\left(t\right)$, was calculated and referred to as $r_{T}$,$\;\;r_{M1}$, and $r_{M2}$, respectively. The stimulus with the higher correlation with the reconstructed speech envelope was detected as the attended speech. For example, if $r_{T}$ was greater than $\;r_{M1}$ and $r_{M2}$, the target speech was detected as the attended speech. The number of trials in which the target stimulus was detected as the attended speech over the total number of trials is referred to as EEG-based speech detection accuracy. Figure 2 summarizes this procedure.

## 3. Results

### 3.1. Behavioral Speech Recognition Score

As one of our goals was to investigate the spatial selective auditory attention in electrical and acoustic hearing, we grouped our CI data into three hearing categories: 1. electrical hearing only (bilateral CIs + unilateral CI with no residual hearing in the contralateral ear + bimodal CI when the HA is off), 2. acoustic hearing only (unilateral CIs with residual hearing in the contralateral ear when CI is off + bimodal CI when CI is off), and 3. electric + acoustic hearing (unilateral CIs with residual hearing in the contralateral ear + bimodal CI). Moving forward, we use EH for the group of electrical hearing only, AH for the group of acoustic hearing only, and EAH for the group of electric + acoustic hearing. Among nine CI subjects, six subjects were in the EH group (five bilateral CI users, one unilateral CI user who had CI in the right ear, and no residual hearing in the left ear), but three subjects were in the EH and EAH group (two unilateral CIs and one bimodal CI).

**Figure 3:**
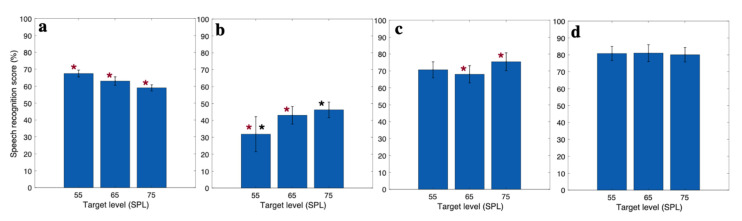
Speech recognition score for (**a**) EH, (**b**) AH, (**c**) EAH CI listeners, and (**d**) NH listeners, (In each plot, asterisks (*) with the same color show the significant difference between the corresponding scores).

The speech recognition scores were calculated as the number of correct words selected by the subjects over the total number of words. The scores are represented in percentage for the EH, AH, and EAH CI users in Figure 3a–c, respectively. In the EH category, the speech recognition score decreased as the target sound level increased in a constant TMR. In contrast, in the AH category, increasing the target speech level resulted in an increased speech recognition score. In the EAH category, the speech recognition score decreased by increasing the target level from 55 dB SPL to 65 dB SPL and increased by increasing the target level from 65 dB SPL to 75 dB SPL. A one-way analysis of variance (ANOVA) was conducted on the speech recognition score with a factor of the target level for each group. The results showed that there was a significant main effect of the target level in EH (F(2,2547) = 18.22, *p* < 0.01), AH (F(2,447) = 9.04, *p* < 0.01), and EAH (F(2,447) = 3.8, *p* = 0.02). A pairwise comparison with Bonferroni adjustment showed that the speech recognition score for the EH category was significantly higher when the target level was at 55 dB SPL than that when the target level was at 65 and 75 dB SPL, and the speech recognition score was significantly higher when the target level was at 65 dB SPL than that when the target level was at 75 dB SPL. The pairwise comparison for the speech recognition score in the AH category showed that the speech recognition score increased by increasing the target level. The speech recognition score at 55 dB SPL target was significantly lower than the speech recognition score at 65 and 75 dB SPL, but there was no significant difference between the speech recognition score when the target level was 65 and 75 dB SPL. For the EAH category, the speech recognition score at the 65 dB SPL target was significantly lower than that at the 75 dB SPL target. The asterisk (*) in Figure 3 shows the significant difference between the speech recognition score at different target levels. The speech recognition scores for the NH group were nearly equal across all three speech levels (Figure 3d). The ANOVA test showed no significant effect of the target speech level on the speech recognition score for NH subjects.

### 3.2. EEG Based Speech Detection Accuracy

**Figure 4:**
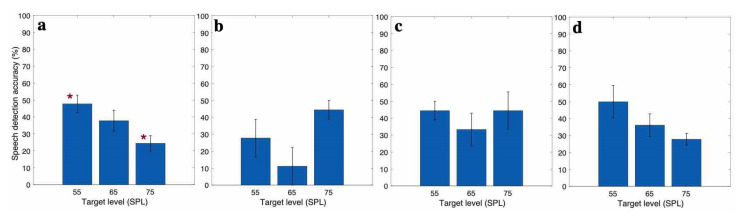
Speech detection accuracy for CI subjects in the case of (**a**) EH, (**b**) AH, and (**c**) EAH CI listeners and (**d**) NH listeners, (asterisks (*) show the significant difference between the corresponding accuracies).

With the individualized decoder for each subject, the EEG data recorded in the three-speaker cocktail party experiment was decoded to find the attended speech, as explained in Section 2.3. The speech detection accuracy for different target speech levels is presented in Figure 4a for CI subjects in the EH category, Figure 4b for AH category, and Figure 4c for EAH category. The statistical analysis ANOVA showed a significant effect of the target level on target detection accuracy in EH category. As Figure 4a depicts, by increasing the target level (while keeping the TMR at a fixed level), the speech detection accuracy decreased. The pairwise comparison with Bonferroni adjustment for the EH category showed that the speech detection accuracy at 55 dB SPL target was significantly higher than that at the 75 dB SPL target. The asterisk (*) in Figure 4 shows the significant difference in the speech detection accuracy between different target levels. There was no effect of target level on the speech detection accuracy in the AH and EAH categories. The speech detection accuracy for different target levels is presented in Figure 4d for NH subjects. The results show a decreasing pattern of the speech detection accuracy as the target level increased. Despite the pattern, the ANOVA showed no significant effect of the target level on the speech detection accuracy for NH subjects.

### 3.3. Case Report for Individual Subjects

Considering significant variability in hearing modality and background among our CI subjects and the small sample size, we are presenting the analysis outcome on a “case-by-case” basis. Each CI subject’s level-dependent spatial hearing patterns were individually demonstrated to be associated with their hearing characteristics. Subjects CI 5 and CI 8 did not complete the physiological part of the experiment and are not included in the analysis.

#### 3.3.1. Subject CI 1

**Figure 5:**
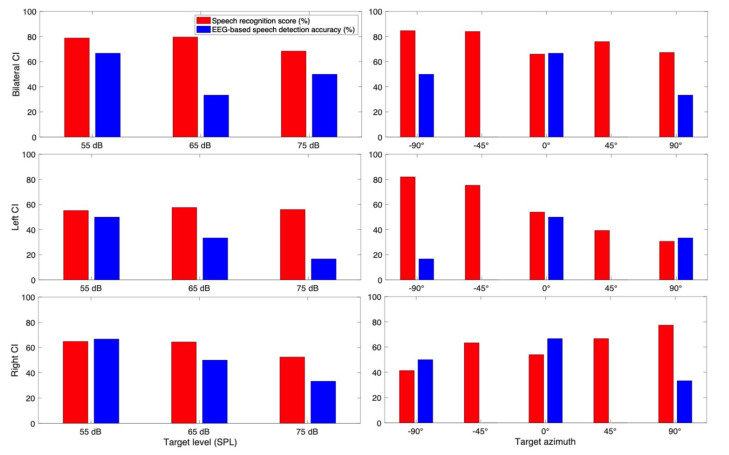
Speech recognition score and speech detection accuracy from EEG for CI 1.

CI 1 (75 years old male) is a bilateral CI user with no residual hearing in either ear (Figure 5). He showed better speech recognition scores with bilateral hearing compared to those with unilateral hearing with one of his CIs off. His scores were likely to be higher at the lower target speech level (55 dB SPL) compared to the higher target speech level (75 dB SPL). His spatial hearing scores were higher when speech was presented in the direction where his CI was on.

Neural outcomes represented that higher neural tracking accuracy was associated with a lower conversational level of the speech in both unilateral CI conditions. Speech detection of neural tracking was the highest when the target speech was presented from 0° azimuth. This neural detection accuracy pattern is somewhat discrepant with his behavioral outcome, where the evidence of directional benefits of CI is provided.

#### 3.3.2. Subject CI 2

**Figure 6:**
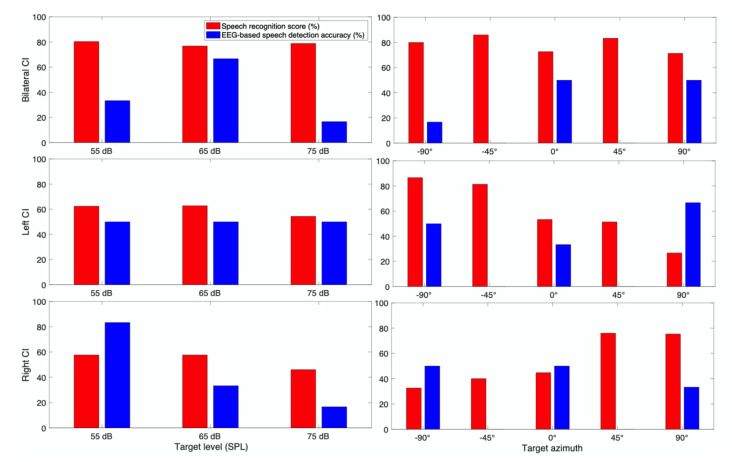
Speech recognition score and speech detection accuracy from EEG for CI 2.

CI 2 (40 years old female) is a prelingually deafened CI user in both ears (Figure 6). Her speech recognition scores were nearly equal across the levels for bilateral conditions. On the other hand, scores for the 75 dB SPL condition were significantly lower than scores for 55 and 65 dB SPL conditions when only one CI was used. A consistent trend of directional benefits was observed when either left or right CI was on. In her overall neural outcomes, clear patterns of level and directionality were not found.

#### 3.3.3. Subject CI 3

**Figure 7:**
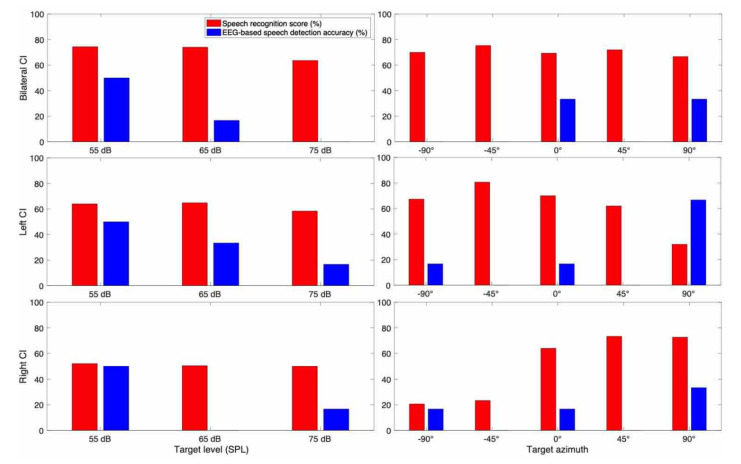
Speech recognition score and speech detection accuracy from EEG for CI 3.

CI 3 (68 years old male) is a post-lingually deafened bilateral CI (Figure 7). In the behavioral speech recognition test, he showed the advantage of bilateral hearing over unilateral hearing. There was a level effect in which slightly higher scores were shown for the lower target speech level compared to the higher target speech level. A clear trend of the directional benefit of CI is also observed.

In neural detection accuracy, higher EEG-based speech detection accuracies with lower speech levels and closer directions to the target speech azimuth were observed. There were few exceptions including the left CI condition showing better neural accuracy at 90° (right), and the right CI condition showing a 0 point of neural accuracy at 65 dB SPL.

#### 3.3.4. Subject CI 4

**Figure 8:**
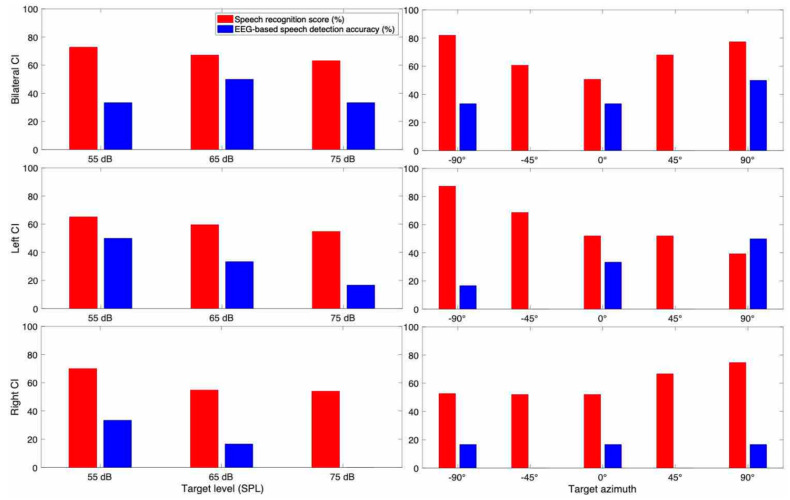
Speech recognition score and speech detection accuracy from EEG for CI 4.

CI 4 (25 years old female) showed the advantage of bilateral hearing over unilateral hearing at all three sound levels (Figure 8). By increasing the sound level, the speech recognition score decreased. The spatial speech recognition score was higher at the azimuths closer to the direction of the activated CI.

EEG results showed that except for the bilateral condition, speech detection accuracy decreased as the target level increased. The pattern of speech detection accuracy based on different target azimuths is not consistent with behavioral results. We expected to have higher speech detection accuracy at the azimuths closer to the amplification side, but the results show a decreasing pattern of speech detection accuracy as the target went from 90° to the −90°, in the bilateral and left CI condition. The speech detection accuracy is less than chance level (33%) in the right CI condition at all target azimuths.

#### 3.3.5. Subject CI 6

**Figure 9:**
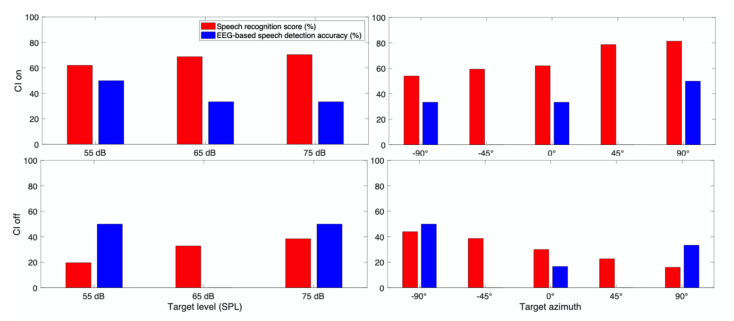
Speech recognition score and speech detection accuracy from EEG for CI 6.

CI 6 (67 years old male) was a unilateral (right) CI user with 70 dB HL of pure tone average (PTA) in his contralateral ear (Figure 9). The PTA is calculated by averaging the hearing threshold at 500 Hz, 1 kHz, and 2 kHz frequencies. He receives a significant amount of electrical energy via his right ear CI and receives some amount of acoustic energy through his left ear in conditions where CI is either on or off. Unlike the EH group (e.g., CI 1, CI 2, CI 3, and CI 4), speech recognition scores for this subject increased by increasing the speech level in both CI-on and CI-off conditions, which shows taking advantage of residual hearing in higher sound levels. His spatial hearing outcomes in CI-on condition clearly indicated CI benefits in the right ear showing higher scores at the conditions where speeches were presented from azimuths closer to the CI side.

The level effect shown in the results of the EEG test was different from that of the behavioral test. Spatial hearing outcomes showed some indications of CI benefits for the right ear in CI-on condition, but they were not very systematic in the CI-off condition. In the CI-off condition (where he relies only on the left ear), we expected to have higher speech detection accuracy at 0° compared to 90°. However, in Figure 9, the speech detection accuracy at 90° is higher than that at 0°.

#### 3.3.6. Subject CI 7

**Figure 10:**
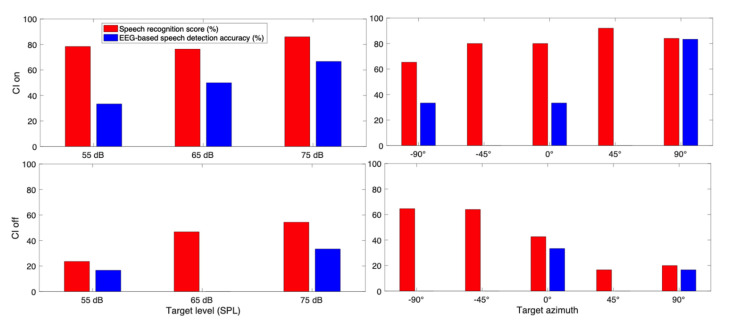
Speech recognition score and speech detection accuracy from EEG for CI 7.

CI 7 (67 years old male) was a unilateral (right) CI user with 71.6 dB HL of PTA in his contralateral ear (Figure 10). Thus, both CI 6 and CI 7 are similar in terms of their degree of residual hearing and hearing modality. His speech recognition score was higher at a higher presentation level of 75 dB in the CI-on condition as well as the CI-off condition. Consistent with other individuals’ spatial hearing patterns, his spatial hearing tends to be better in the direction that provides him with better audibility.

Overall, neural tracking of CI 7 appeared to be consistent with his behavioral pattern when his CI was on. A few exceptions, however, were found in the CI-off condition (EEG-based speech detection accuracy for 65 dB SPL was lower than that for 55 dB SPL, and at −90° it was lower than that at either 0° or 90°).

#### 3.3.7. Subject CI 9

**Figure 11:**
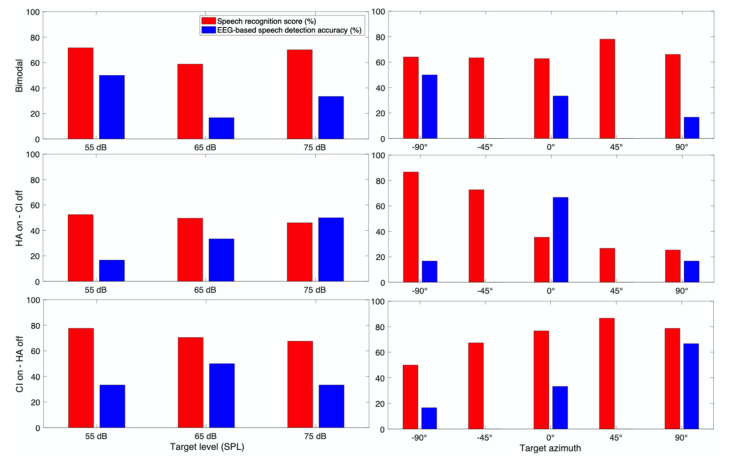
Speech recognition score and speech detection accuracy from EEG for CI 9.

CI 9 (77 years old male) was the sole bimodal subject of our study who used CI in his right ear and HA in his left ear (Figure 11). These types of subjects are known to receive a great benefit from their acoustic hearing via HA as well as CI. Our behavioral results showed that his perceptual performance was higher when using CI compared to HA. Bimodal benefits were shown in 55 dB SPL and 75 dB SPL conditions, but not in 65 dB SPL conditions. When CI was on, higher score was obtained at the softer target speech level, but this effect disappeared with the addition of acoustic hearing. His spatial hearing performance followed the general trend, showing higher scores in the direction the amplification was placed.

EEG outcome was not consistent with behavioral outcomes showing some nonsystematic patterns as a function of the speech level and target direction. In contrast with the behavioral results, in HA-on condition, the speech recognition score increased by increasing the sound level. There is a clear directional pattern in CI-on condition, in which higher EEG-based speech detection accuracy was acquired at the azimuths closer to the hearing side.

## 4. Discussion

Our behavioral test results presented no statistical difference in NHs’ speech recognition score under different conversational sound levels. However, we found that sound level significantly affected CIs’ speech recognition score. The electrical dynamic range of CIs differs from the dynamic range of NH; thus, the loudness that CI listeners perceive may be different from that which NH listeners perceive. It is reasonable to assume that input dynamic range for NH would be approximately 120 dB, considering the loudness comfort NH listeners generally show. However, CIs substantially compress the acoustic dynamic range into a much narrower electrical dynamic range in the system. The electrical dynamic range varies from 10 to 80 dB depending on the signal processing strategy and CI manufacturer [30]. We infer that the compression applied at higher levels may distort the speech recognition in CI users, which negatively affects their spatial selective auditory attention.

In the case of electrical hearing only (EH), the speech recognition score decreased by increasing the sound level. Some studies showed that in a quiet condition, CI listeners’ speech recognition improves by increasing the speech level [31]. However, our previous studies showed that increasing the speech level in the presence of noise at a constant SNR degrades the perceived quality of speech by CI listeners [32,33]. It is generally reported that CI users are more susceptible to noise than NH listeners when recognizing speech. We conclude that in spatial hearing, it is more difficult for CI listeners to suppress the higher masker level accompanied with the increased target level, which resulted in reduced spatial selective auditory attention in the CI group. The CI listeners’ data in the acoustic-hearing-only (EH) category (unilateral CI users with residual hearing in their non-implanted ear and bimodal CI user when their CI was off) showed an improvement in their spatial selective auditory attention with an increase in the sound level. It can be speculated that the negative effect of increased masker levels is less than the positive effect of increased target levels in the less audible listening situation. The electrical hearing seems more vulnerable to the amount of noise even at the same TMR. In other words, the negative effect of increased maskers’ level is perceptually greater than the advantage of the higher audibility of the target. In the case of electric + acoustic hearing (EAH), the results show that the spatial selective auditory attention with target level at 55 dB SPL relies more on electrical hearing (see Figure 3c). By increasing the target level to 65 dB SPL, electrical hearing degraded, while the improvement in the acoustic hearing was not enough to compensate the electrical hearing degradation. Therefore, it resulted in decreased spatial selective auditory attention. Increasing the target level to 75 dB SPL improved the acoustic hearing, which resulted in listeners relying on acoustic hearing rather than electrical hearing.

To see the overall trend of spatial hearing ability according to the different target and masker azimuth, we also examined each individual CI listener results as shown in Figure 5, Figure 6, Figure 7, Figure 8, Figure 9, Figure 10 and Figure 11. As expected, the results showed higher speech recognition scores at the target azimuths that were closer to the side of amplification.

The group mean average for the electrophysiological results was consistent with the behavioral results. For NH listeners, although the EEG-based speech detection accuracy decreased with increasing sound level, the effect of the sound level was not statistically significant. The effect of the sound level was significant for CIs in the EH category and consistent with their behavioral results. There was no significant effect of level on EEG-based speech detection accuracy for CIs in the AH and EAH categories. We should mention that the number of trials included in the electrophysiological experiment was much less than the trials included in the behavioral experiment. Due to the time-demanding nature of the EEG test, we had less repetition in trials for each condition. In addition, only the data from three subjects were included in the AH and EAH groups. Thus, we may argue that the data collected for these groups were not enough to show any significant effect of the sound level.

For both NH and CI listeners, the EEG-based speech detection accuracy at some speech levels and azimuths was around the chance level (33%) in the electrophysiological experiment. One reason may be the difficulty of the task, as subjects were required to attend to the target passage and suppress two competing maskers. It also may suggest that the auditory attention detection approach used in this study is not robust enough to detect the attended speech in the presence of two competing speeches. In previous studies conducted to detect the attended speech through neural response [14,23,24,25,34,35], the target sound was detected in two speaker cocktail party scenarios (one speaker as a target and one speaker as a masker). In real-world scenarios, NH listeners are able to focus on the target speech in the presence of several sources of noise. In the current paper, we examined the neural entrainment to the speech envelope in a three-speaker cocktail party scenario. The linear model used in this study was not able to detect the auditory attention at some of the sound levels, probably due to the increased number of maskers. Other methods, including non-linear models, should be investigated to better understand the selective auditory attention in the presence of multiple maskers.

Individual subjects’ outcomes were independently demonstrated in the case study report to better present the limited sample size group analysis. Individual factors associated with diverse hearing conditions have influenced the outcomes that were not uniform across the subjects. This study would provide a preliminary outcome and become a steppingstone to control such individual variabilities in typical CI users. The variability control is necessary to facilitate the data analysis in future studies.

The major limitations of this study were the small sample size and unbalanced number of subjects in each hearing mode group. These were caused by a small available local pool of CI population with different kinds of assisted/impaired hearing modes. The results from the small sample size in each group may not necessarily be generalized to the specific group. Additionally, the unbalanced number of participants in each group may result in a biased conclusion. More extensive investigations with multicenter studies should be conducted in future research to provide more concrete conclusions. The other limitation that can be improved in future studies is the lack of balance between the number of behavioral and electrophysiological trials. As was mentioned in the Method section, the number of EEG trials has been reduced to avoid human fatigue. For future studies, the electrophysiological experiment should be extended to several days, which would allow more repetition for each sound level and spatial configuration.

## 5. Conclusions

This study examined the contribution of speech level on the spatial selective auditory attention in acoustic and electrical hearing. The results for NH listeners showed that, at a fixed TMR, changing the level of the target speech in the conversational-level range will not affect selective auditory attention. However, the results for CI listeners with “electrical hearing only” showed that increasing the speech level resulted in decreased speech recognition scores. On the other hand, in the group of “acoustic hearing only”, increasing the speech level resulted in the increased speech recognition score. Due to the limited samples in each hearing group, the case-by-case results are reported. Our results suggest that the loudness control should be ideally based on the listener’s hearing mode. This work should be considered as a preliminary study attempting to investigate CI users’ selective auditory attention in the behavioral and electrophysiological approach and more extensive and robust investigation should be made for future studies.

## Figures and Tables

**Table 1:** Demographic information of CI listeners.

Subject Number	Age (years)	Gender	CI Ear	CI Model	CI Use (years)	Speech Processing Strategy	Duration of HL (years)	Pure Tone Average of 500, 1000, and 2000 Hz (dB HL)	Etiology
Bilateral CI									
1	75	Male	Both	Medel/Sonnet	7	FS4	33	Right: NR Left: NR	Meniere’s Disease
2	40	Female	Both	Medel/Sonnet	4	FS4	38.5	Right: NR Left: NR	unknown
3	68	Male	Both	Medel/Sonnet	10	FS4	38	Right: NR Left: NR	unknown
4	25	Female	Both	Cochlear/Nucleus 6	21	ACE	25	Right: NR Left: NR	unknown
5 *	77	Female	Both	Medel/Sonnet	70	FS4	6	Right: NR Left: NR	meningitis
Unilateral CI									
6	67	Male	Right	Cochlear/Nucleus 6	11	ACE	11	Right: NR Left: 70	Noise induced
7	67	Male	Right	Medel/Sonnet	10	FS4	24	Right: NR Left: 71.6	Meniere’s Disease
8 *	24	Female	Right	Cochlear/Nucleus 7	1	ACE	23	Right: NR Left: NR	Genetic
Bimodal hearing									
9	77	Male	Right	Medel/Sonnet	2	FS4	20	Right: 82.5 Left: 66.6	Noise induced

* Subject 5 and 8 from CI listeners and two of the NH listeners were not able to participate in EEG recording.

**Table 2:** Matrix sentences.

Name	Verb	Number	Adjective	Noun
Jane	Took	Two	New	Toys
Gene	Gave	Three	Old	Hats
Pat	Lost	Four	Big	Shoes
Bob	Found	Five	Small	Cards
Sue	Bought	Six	Red	Pens
Mike	Sold	Seven	Blue	Socks
Lynn	Held	Eight	Cold	Bags
Jill	Saw	Nine	Hot	Gloves

## Data Availability

The data used to support the findings of this study are available from the corresponding author upon request.

## Appendix A

To detect the auditory attention in the cocktail party scenario, we reconstructed the attended speech envelope from an EEG signal. We needed a decoder that maps the speech envelope to the corresponding EEG signal. Individualized decoder for each subject was trained using the presented speech and recorded EEG signal in the training session. We used mTRF toolbox (version 1.5) [36] to train the decoder and reconstruct the attended speech envelope from the neural response.

The speech envelopes of the stimuli were extracted using the Hilbert transform, and band pass was filtered to 0.3–30 Hz using MATLAB filtfilt function. To decrease the processing time, speech envelopes were downsampled to 128 Hz and normalized to a range of values between 0.0 and 1.0.

After preprocessing of the EEG signal recorded from 64 channels and removing the artifacts using independent component analysis (ICA), EEG waves were band-pass filtered to 0.3–30 Hz using Fieldtrip toolbox [37]. The filtered EEGs were downsampled to 128 Hz and normalized to (0–1). All the implementations were in MATLAB 2018b.

Suppose that the linear mapping from the instantaneous neural response r(t,n), sampled at times t=1,…,T and at channel n, to the speech envelope s(t) is represented as d(τ,n), which is the decoder that integrates the neural response over a range of time lags τ. The linear convolution equation corresponding to this system can be expressed as:

(A1) $$\hat{s}\left(t\right)=\underset{n}{\sum }\underset{\tau }{\sum }r\left(t+\tau ,n\right)d\left(\tau ,n\right),$$

In Equation (A1), $\hat{s}\left(t\right)$ is the reconstructed speech envelope. The cost function to design the decoder is the MSE between s(t) and $\hat{s}\left(t\right)$.

(A2) $$\min\varepsilon \left(t\right)=\underset{t}{\sum }{\left[s\left(t\right)-\hat{s}\left(t\right)\right]}^{2},$$

which results in the following formula for decoder:

(A3) $$d={\left(R^{T}R\right)}^{-1}R^{T}s,$$

In the above equation, R represents the lagged time series of neural response matrix r. If $R_{c}$ contains all time lags $\left[\tau_{min},\tau_{max}\right]$ for channel c, then:

(A4) $$R=\left[R_{1}\;R_{2}\;\cdots \;R_{N}\right],$$

where:

(A5) $$R_{c}=[\begin{array}{ccccccc} r\left(1-\tau_{min},c\right) & r\left(-\tau_{min},c\right) & \cdots & r\left(1,c\right) & 0 & \cdots & 0\\ \vdots & \vdots & \cdots & \vdots & r\left(1,c\right) & \cdots & \vdots \\ \vdots & \vdots & \cdots & \vdots & \vdots & \cdots & 0\\ \vdots & \vdots & \cdots & \vdots & \vdots & \cdots & r\left(1,c\right)\\ r\left(T,c\right) & \vdots & \cdots & \vdots & \vdots & \cdots & \vdots \\ 0 & r\left(t,c\right) & \cdots & \vdots & \vdots & \cdots & \vdots \\ \vdots & 0 & \cdots & \vdots & \vdots & \cdots & \vdots \\ \vdots & \vdots & \cdots & \vdots & \vdots & \cdots & \vdots \\ 0 & 0 & \cdots & r\left(T,c\right) & r\left(T-1,c\right) & \cdots & r\left(T-\tau_{max},c\right) \end{array}],$$

To avoid overfitting of the model to the training data, Tikhonov regularization is applied on the optimization problem of Equation (A2), which results in:

(A6) $$d={\left(R^{T}R+\lambda I\right)}^{-1}R^{T}s,$$

In the above equation, λ is the regularization parameter. To find the best λ, we used leave-one-out cross-validation. The training data (speech stimuli and corresponding EEG signal collected in training session) were split to K trials. The preliminary decoders constructed from single trials would be ${\tilde{d}}_{k}={\left(R_{\left(k\right)}^{T}R_{\left(k\right)}+\lambda I\right)}^{-1}R_{\left(k\right)}^{T}S_{\left(k\right)}$, where $R_{\left(k\right)}$ and $S_{\left(k\right)}$ are the EEG response and stimuli of trial k (see Equation (A6)). The preliminary decoders from all trials except trial k were averaged to define decoder $d_{k}$, which decodes trial k.

(A7) $$d_{k}=\frac{1}{K-1}\overset{K}{\underset{\begin{array}{c} i=1\\ i\neq k \end{array}}{\sum }}{\tilde{d}}_{i}\;\;\;\;\;\;\;\;\;\;\;\;\;\;k=1,\cdots ,\;K$$

Using the decoders defined by Equation (A7) for each trial, the speech envelopes were reconstructed. The Pearson correlation between the reconstructed speech envelope and the original speech envelope is a criterion to evaluate the accuracy of the decoder. The λ that resulted in maximum correlation averaged across K trials was selected to train the decoder. Here, we split the training data of each subject into 20 trials and examined a range of $\lambda =\left\{10^{-3},10^{-2},\cdots ,10^{11}\right\}$.

Different CI users receive different signals from their CIs, based on the processing strategies and specific artifacts caused by different RF transmission systems. Therefore, we selected an individualized λ value for each CI user using each individual’s training data. For NH listeners, we selected a common λ value of $10^{5}$. The λ for each CI subject is shown in Table A1.

**Table A1:** Selected *λ* for CI subjects.

Subject Number	CI-1	CI-2	CI-3	CI-4	CI-6	CI-7	CI-9
***λ***	10^1^	10^5^	10^1^	10^5^	10^−1^	10^3^	10^1^

## References

[1] Shannon R.V., Zeng F.G., Kamath V., Wygonski J., Ekelid M. (1995). Speech recognition with primarily temporal cues. Science.

[2] Shinn-cunningham B.G., Best V. (2008). Selective attention in normal and impaired hearing. Trends Amplif..

[3] Mackersie C.L., Prida T.L., Stiles D. (2001). The role of sequential stream segregation and frequency selectivity in the perception of simultaneous sentences by listeners with sensorineural hearing loss. J. Speech Lang. Hear. Res..

[4] Marrone N., Mason C.R., Kidd G. (2008). The effects of hearing loss and age on the benefit of spatial separation between multiple talkers in reverberant rooms. J. Acoust. Soc. Am..

[5] Best V., Marrone N., Mason C.R., Kidd G., Shinn-Cunningham B.G. (2008). Effects of Sensorineural Hearing Loss on Visually Guided Attention in a Multitalker Environment. J. Assoc. Res. Otolaryngol..

[6] Dai L., Best V., Shinn-Cunningham B.G. (2018). Sensorineural hearing loss degrades behavioral and physiological measures of human spatial selective auditory attention. Proc. Natl. Acad. Sci. USA.

[7] Murphy J., Summerfield A.Q., O’Donoghue G.M., Moore D.R. (2011). Spatial hearing of normally hearing and cochlear implanted children. Int. J. Pediatr. Otorhinolaryngol..

[8] Mok M., Galvin K., Dowell R.C., McKay C.M. (2007). Spatial Unmasking and Binaural Advantage for Children with Normal Hearing, a Cochlear Implant and a Hearing Aid, and Bilateral Implants. Audiol. Neurotol..

[9] Bennett E.E., Litovsky R.Y. (2020). Sound Localization in Toddlers with Normal Hearing and with Bilateral Cochlear Implants Revealed Through a Novel “Reaching for Sound” Task. J. Am. Acad. Audiol..

[10] Wright H.M., Bulla W., Tarr E.W. (2019). Spatial release from masking and sound localization using real-time sensorineural hearing loss and cochlear implant simulation. J. Acoust. Soc. Am..

[11] Winn M.B., Won J.H., Moon I.J. (2016). Assessment of spectral and temporal resolution in cochlear implant users using psychoacoustic discrimination and speech cue categorization. Ear Hear..

[12] Seebacher J., Franke-Trieger A., Weichbold V., Zorowka P., Stephan K. (2019). Improved interaural timing of acoustic nerve stimulation affects sound localization in single-sided deaf cochlear implant users. Hear. Res..

[13] Francart T., Brokx J., Wouters J. (2008). Sensitivity to Interaural Level Difference and Loudness Growth with Bilateral Bimodal Stimulation. Audiol. Neurotol..

[14] Arbogast T.L., Mason C.R., Kidd G. (2002). The effect of spatial separation on informational and energetic masking of speech. J. Acoust. Soc. Am..

[15] Glyde H., Hickson L., Cameron S., Dillon H. (2011). Problems hearing in noise in older adults: A review of spatial processing disorder. Trends Amplif..

[16] Best V., Marrone N., Mason C.R., Kidd G. (2012). The influence of non-spatial factors on measures of spatial release from masking. J. Acoust. Soc. Am..

[17] Strelcyk O., Dau T. (2009). Relations between frequency selectivity, temporal fine-structure processing, and speech reception in impaired hearing. J. Acoust. Soc. Am..

[18] Smoski W.J., Trahiotis C. (1986). Discrimination of interaural temporal disparities by normal-hearing listeners and listeners with high-frequency sensorineural hearing loss. J. Acoust. Soc. Am..

[19] Hawkins D.B., Wightman F.L. (1980). Interaural Time Discrimination Ability of Listeners with Sensorineural Hearing Loss. Int. J. Audiol..

[20] Ching T.Y.C., Van Wanrooy E., Dillon H., Carter L. (2011). Spatial release from masking in normal-hearing children and children who use hearing aids. J. Acoust. Soc. Am..

[21] Gallun F.J., Diedesch A.C., Kampel S.D., Jakien K.M. (2013). Independent impacts of age and hearing loss on spatial release in a complex auditory environment. Front. Neurosci..

[22] Akbarzadeh S., Lee S., Chen F., Tan C.-T. The effect of perceived sound quality of speech in noisy speech perception by normal hearing and hearing impaired listeners. Proceedings of the 2019 41st Annual International Conference of the IEEE Engineering in Medicine and Biology Society (EMBC).

[23] Mesgarani N., Chang E.F. (2012). Selective cortical representation of attended speaker in multi-talker speech perception. Nat. Cell Biol..

[24] O’Sullivan J.A., Power A.J., Mesgarani N., Rajaram S., Foxe J., Shinn-Cunningham B.G., Slaney M., Shamma S.A., Lalor E.C. (2015). Attentional Selection in a Cocktail Party Environment Can Be Decoded from Single-Trial EEG. Cereb. Cortex.

[25] Horton C., Srinivasan R., D’Zmura M. (2014). Envelope responses in single-trial EEG indicate attended speaker in a ‘cocktail party’. J. Neural Eng..

[26] Vanthornhout J., Decruy L., Wouters J., Simon J., Francart T. (2018). Speech Intelligibility Predicted from Neural Entrainment of the Speech Envelope. J. Assoc. Res. Otolaryngol..

[27] Kidd G., Best V., Mason C.R. (2008). Listening to every other word: Examining the strength of linkage variables in forming streams of speech. J. Acoust. Soc. Am..

[28] Cox R.M., Genevieve C.G., Alexander C. (1987). Development of the Connected Speech Test (CST).pdf. Ear Hear..

[29] Cox R.M., McDaniel D.M. (1989). Development of the speech intelligibility rating (SIR) test for hearing aid comparisons. J. Speech Lang. Hear. Res..

[30] Zeng F.-G., Grant G., Niparko J., Galvin J., Shannon R., Opie J., Segel P. (2002). Speech dynamic range and its effect on cochlear implant performance. J. Acoust. Soc. Am..

[31] Firszt J.B., Holden L.K., Skinner M.W., Tobey E.A., Peterson A., Gaggl W., Runge-Samuelson C.L., Wackym P.A. (2004). Recognition of Speech Presented at Soft to Loud Levels by Adult Cochlear Implant Recipients of Three Cochlear Implant Systems. Ear Hear..

[32] Akbarzadeh S., Lee S., Singh S., Tuan-Tan C. Implication of speech level control in noise to sound quality judgement. Proceedings of the 2018 Asia-Pacific Signal and Information Processing Association Annual Summit and Conference (APSIPA ASC).

[33] Akbarzadeh S., Lee S., Chen F., Tuan-Tan C. (2021). The effect of speech and noise levels on the quality perceived by cochlear implant and normal hearing listeners. Speech Commun..

[34] Biesmans W., Das N., Francart T., Bertrand A. (2017). Auditory-Inspired Speech Envelope Extraction Methods for Improved EEG-Based Auditory Attention Detection in a Cocktail Party Scenario. IEEE Trans. Neural Syst. Rehabil. Eng..

[35] Mirkovic B., Debener S., Jaeger M., De Vos M. (2015). Decoding the attended speech stream with multi-channel EEG: Implications for online, daily-life applications. J. Neural Eng..

[36] Crosse M.J., Di Liberto G.M., Bednar A., Lalor E.C. (2016). The Multivariate Temporal Response Function (mTRF) Toolbox: A MATLAB Toolbox for Relating Neural Signals to Continuous Stimuli. Front. Hum. Neurosci..

[37] Oostenveld R., Fries P., Maris E., Schoffelen J.-M. (2010). FieldTrip: Open Source Software for Advanced Analysis of MEG, EEG, and Invasive Electrophysiological Data. Comput. Intell. Neurosci..

